# Ascorbic Acid Protects against Hypertension through Downregulation of ACE1 Gene Expression Mediated by Histone Deacetylation in Prenatal Inflammation-Induced Offspring

**DOI:** 10.1038/srep39469

**Published:** 2016-12-20

**Authors:** Jing Wang, Na Yin, Youcai Deng, Yanling Wei, Yinhu Huang, Xiaoyun Pu, Li Li, Yingru Zheng, Jianxin Guo, Jianhua Yu, Xiaohui Li, Ping Yi

**Affiliations:** 1 Department of Obstetrics and Gynecology, Research Institute of Surgery, Daping Hospital, Third Military Medical University, Chongqing, China; 2 Institute of Materia Medica, College of Pharmacy, Third Military Medical University, Chongqing, China; 3 Department of Gastroenterology, Research Institute of Surgery, Daping Hospital, Third Military Medical University, Chongqing, China; 4 The Ohio State University Comprehensive Cancer Center, Columbus, OH 43210, US

## Abstract

Hypertension is a major risk factor for cardiovascular and cerebrovascular disease. Prenatal exposure to lipopolysaccharide (LPS) leads to hypertension in a rat offspring. However, the mechanism is still unclear. This study unraveled epigenetic mechanism for this and explored the protective effects of ascorbic acid against hypertension on prenatal inflammation-induced offspring. Prenatal LPS exposure resulted in an increase of intrarenal oxidative stress and enhanced angiotensin-converting enzyme 1 (*ACE1*) gene expression at the mRNA and protein levels in 6- and 12-week-old offspring, correlating with the augmentation of histone H3 acetylation (H3AC) on the *ACE1* promoter. However, the prenatal ascorbic acid treatment decreased the LPS-induced expression of *ACE1*, protected against intrarenal oxidative stress, and reversed the altered histone modification on the *ACE1* promoter, showing the protective effect in offspring of prenatal LPS stimulation. Our study demonstrates that ascorbic acid is able to prevent hypertension in offspring from prenatal inflammation exposure. Thus, ascorbic acid can be a new approach towards the prevention of fetal programming hypertension.

Essential Hypertension (EH) is an important risk factor for cardiovascular diseases, and markedly impairs human health and life^1^. It is a frequent, chronic, age-related disorder that is affected by an interaction of genetic and environmental factors^2^. Substantial epidemiological investigations suggest that fetal environments during pregnancy has important effects on blood pressure and hypertension in adults^3^, and hypertension in adult is programmed by an adverse fetal environment *in utero*^4^. Maternal inflammation exposure is one of the most common events in pregnant women who suffer from some inflammatory diseases. Several previous studies indicate that prenatal inflammation exposure is highly associated with adult hypertension in the offspring^5^,^6^,^7^,^8^. Lipopolysaccharide (LPS) is a toxic component of cell walls of gram-negative bacteria, often used as a nonspecific immuno-inflammatory stimulant to mimic the bacterial inflammatory response^9^. The previous studies from our group have shown that prenatal exposure to LPS leads to hypertension in offspring, increased activity of the intrarenal renin-angiotensin system (RAS), and renal damage in adult offspring rats^9^,^10^. However, the mechanism of abnormal intrarenal RAS in offspring with prenatal inflammation exposure is still unclear.

Pyrrolidine dithiocarbamate (PDTC) is a synthetic antioxidant, as a reputed inhibitor of proinflammatory nuclear transcription factor-κB (NF-κB), and it inhibits NF-κB by preventing inhibitor of NF-κB (IκB) degradation and the translocation of the active form to the nucleus^11^. Various studies have suggested that PDTC can ameliorate angiotensin II-induced inflammatory damage and pulmonary hypertension in rats^12^,^13^, attenuate systolic blood pressure and renal inflammatory response in mineralocorticoid hypertensive rats^14^, alleviate renal interstitial inflammation, and prevent hypertension in spontaneously hypertensive rats^15^. We previously showed that prenatal PDTC treatment can markedly reverse the effect of prenatal LPS exposure on offspring^9^,^10^. However, PDTC has not been used in the clinic. This prompted us to identify a clinically used antioxidant to control prenatal inflammation.

Ascorbic acid (AA) is a hydrophilic antioxidant that can scavenge several radicals. It is safer than PDTC and is broadly used in the clinic. It has been demonstrated to be effective on decreasing fetal malformation rate and diminishing oxidative stress in experimental diabetic pregnancy^16^, protecting against LPS-induced intra-uterine fetal death and reversing LPS-induced intra-uterine growth retardation in mice^17^. However, it’s still largely unknown whether and how AA protects against chronic diseases, such as hypertension, in offspring of prenatal inflammation exposure. In this study, we unraveled epigenetic mechanism for abnormal intrarenal RAS in rat offspring with prenatal LPS exposure and explored the protective effects of AA against hypertension in prenatal inflammation-induced offspring.

## Results

### Effects of prenatal LPS exposure on body weight, blood pressure, and urinary protein in offspring

**Figure 1:**
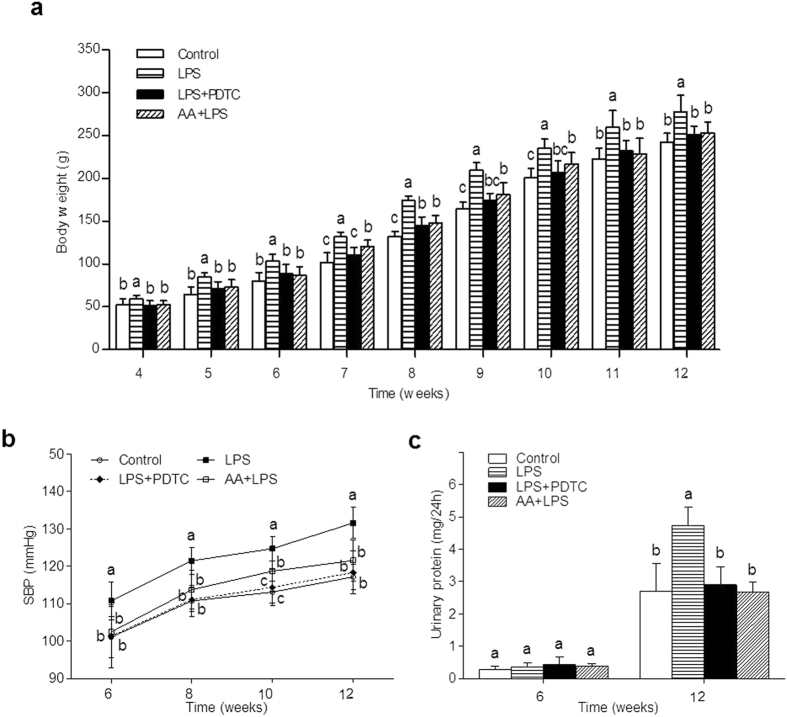
Effects of a prenatal LPS exposure and the PDTC or the AA treatment on body weight, blood pressure, and urinary protein in offspring. The body weight (**a**) of offspring was measured once a week from 4 to 12 weeks old. (n = 8 in each group). Systolic blood pressure (**b**, SBP) was measured by a tail-cuff method in 6, 8, 10 and 12 weeks old offspring. (n = 8 in each group). Urinary protein (**c**) was measured in 6 and 12 weeks old offspring. (n = 5 in each group). Data are expressed as mean ± SD. Different letters means significant differences (*P* < 0.05).

To explore the protective effect of AA on adult offspring of prenatal inflammation exposure, we first determined the blood pressure and body weight. As shown in Fig. 1, the body weight of offspring in the LPS group was significantly higher than control group at the ages of 4–12 weeks (Fig. 1a). The systolic blood pressure (SBP) of offspring in the LPS group was found to be significantly higher than that in the control group from 6 to 12 weeks (Fig. 1b). The urinary protein level did not have significant differences in the offspring of four groups at 6 weeks of age, but was observably higher in the offspring of LPS group than control group at 12 weeks of age (Fig. 1c). Thus, our data showed that after the PDTC or the AA treatment, the body weight, SBP, and urinary protein level of offspring were strikingly reduced (Fig. 1). We found that prenatal AA administration effectively reversed the increased SBP and body weight caused by prenatal inflammation stimulation, which showed comparable effect as the PDTC treatment.

### Effects of prenatal LPS exposure on RAS mRNA and protein expression in renal cortex of offspring

**Figure 2:**
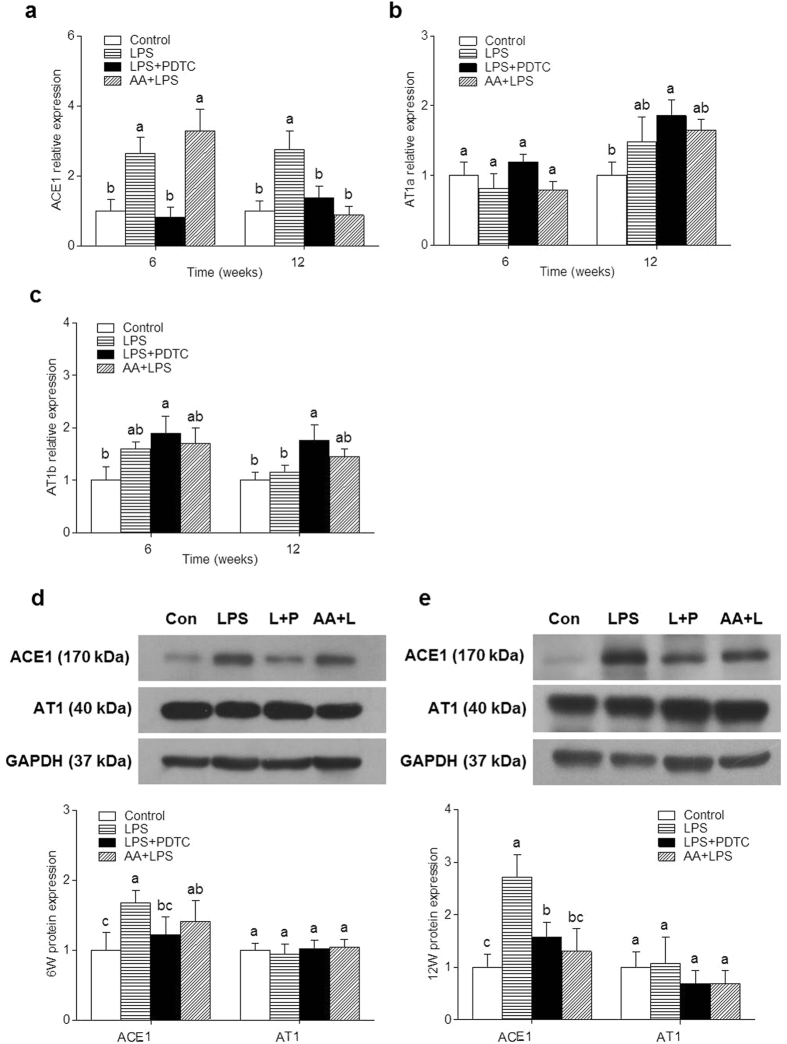
Effects of a prenatal LPS exposure and the PDTC or the AA treatment on renal cortex RAS mRNA and protein expression in offspring. Renal cortex *ACE1* (**a**), *AT1a* (**b**) and *AT1b* (**c**) mRNA expression were determined by real-time PCR in 6 and 12 weeks old offspring, *GAPDH* was taken as internal control. Renal cortex *ACE1* and *AT1* protein expression were assessed by immunoblotting in 6 (**d**) and 12 (**e**) weeks old offspring, *GAPDH* was taken as internal control. Data are expressed as mean ± SD. (n = 6 in each group). Different letters means significant differences (*P* < 0.05).

Compared with controls, the angiotensin-converting enzyme 1 (*ACE1*) mRNA expression in renal cortex showed a significant increase in the offspring of LPS group at 6 and 12 weeks, both the PDTC treatment and the AA treatment could observably inhibit the increase in *ACE1* mRNA expression (Fig. 2a). The angiotensin II type-1a receptor (*AT1a*) and angiotensin II type-1b receptor (*AT1b*) mRNA expression did not have significant changes in the offspring of four groups at 6 and 12 weeks (Fig. 2b,c). In particular, the expression of angiotensin II type-2 receptor (*AT2*) in renal cortex of offspring at 6 and 12 weeks was below the threshold of detection.

The protein expression of *ACE1* in renal cortex of LPS group offspring was significantly higher than that in the control group at 6 and 12 weeks of age, and the PDTC or the AA treatment could markedly reduce the expression (Fig. 2d,e). There was no obvious difference in the angiotensin II type-1 receptor (*AT1*) protein expression from offspring of the four groups at 6 and 12 weeks (Fig. 2d,e).

### Effects of prenatal LPS exposure on oxidative stress in offspring

**Figure 3:**
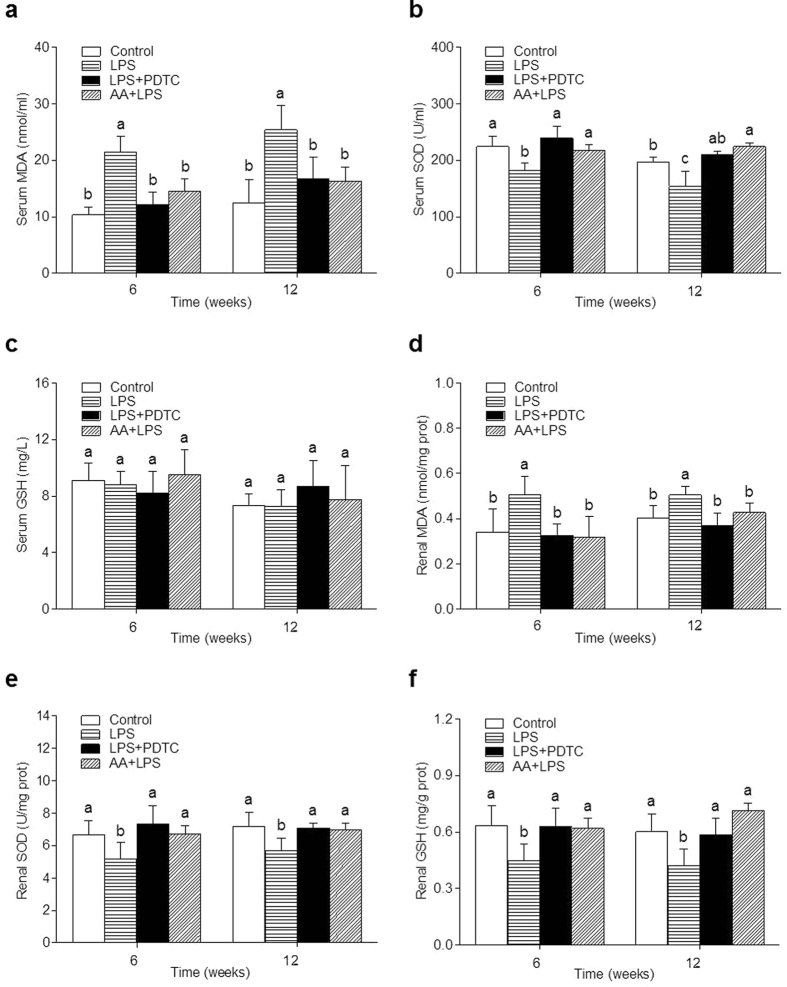
Effects of a prenatal LPS exposure and the PDTC or the AA treatment on oxidative stress in offspring. Serum MDA (**a**) Serum SOD (**b**) Serum GSH (**c**) renal MDA (**d**) renal SOD (**e**) and renal GSH (**f**) were determined in 6 and 12 weeks old offspring. Data are expressed as mean ± SD. (n = 6 in each group). Different letters means significant differences (*P* < 0.05).

The serum and renal malondialdehyde (MDA) levels were significantly higher and superoxide dismutase (SOD) levels were significantly lower in the offspring of LPS group than that in the control group at 6 and 12 weeks of age. Both PDTC and AA treatments observably decreased the MDA levels and increased SOD levels at 6 and 12 weeks (Fig. 3a,b,d and e). The renal glutathione (GSH) level was markedly lower in the offspring of LPS group than control group at 6 and 12 weeks, and both the PDTC and the AA treatment could observably reverse the reduction (Fig. 3f). Of note, the serum GSH level did not have significant differences in the offspring of four groups at 6 and 12 weeks (Fig. 3c).

### Effects of prenatal LPS exposure on DNA methylation and histone acetylation in *ACE1* gene of renal cortex

**Figure 4:**
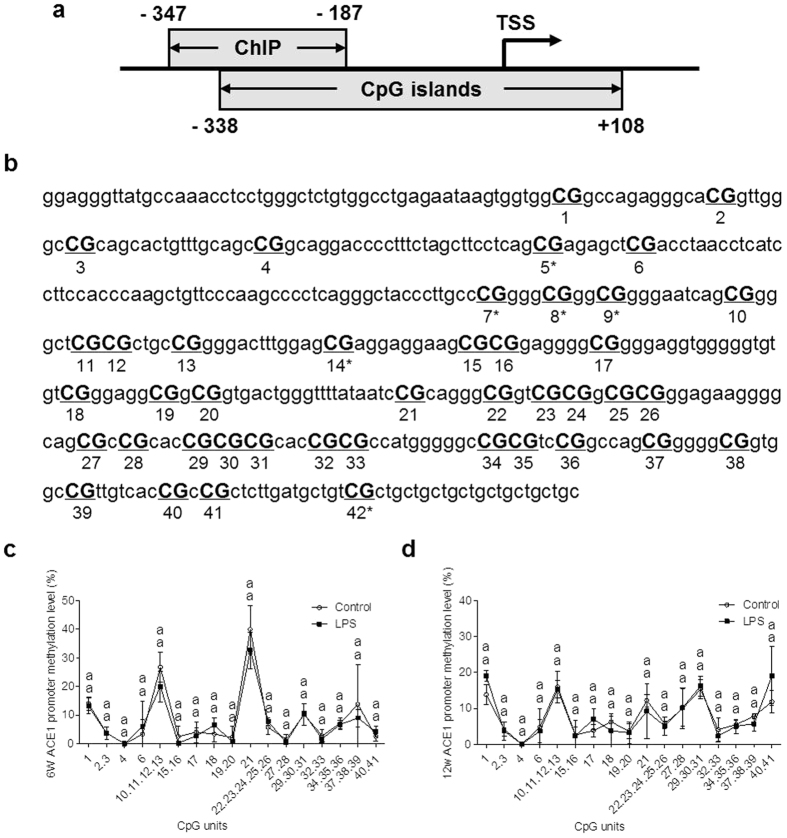
Effects of a prenatal LPS exposure and the PDTC or the AA treatment on renal cortex *ACE1* DNA methylation in offspring. (**a**) The positions of CpG island analysis, and ChIP analysis are shown schematically in which the numbers indicate the nucleotide location starting from transcription start site (TSS; +1). (**b**) is the selected amplicon of methylation analysis and the 42 CpG units. Unit 5*, 7*, 8*, 9*, 14* and 42* cannot be determined because of sequence problem. (**c,d**) Renal cortex *ACE1* CpG island DNA methylation were determined by a MassARRAY Compact MALDI-TOF method in 6 and 12 weeks old offspring. Data are expressed as mean ± SD. (n = 6 in each group). ChIP; Chromatin immunoprecipitation. Different letters means significant differences (*P* < 0.05).

To understand the cause of *ACE1* expression change in renal cortex of adult offspring of prenatal inflammation exposure, we first examined methylation status of the CpG islands of the *ACE1* gene. We analyzed the promoter region, 5′ Un-Translated Region (UTR) and 3′ UTR of *ACE1*. The CpG islands are located in the promoter region of *ACE1* gene. There are 42 CpG units in selected amplicon of the *ACE1* promoter region (Fig. 4b). Of the total CpGs, methylation level of 6 CpG units could not be determined by Sequenom MassARRAY because of a sequencing problem. However, the DNA methylation level of other *ACE1* CpG units in renal cortex did not have significant differences between the offspring of control and LPS group at 6 and 12 weeks (Fig. 4c,d).

**Figure 5:**
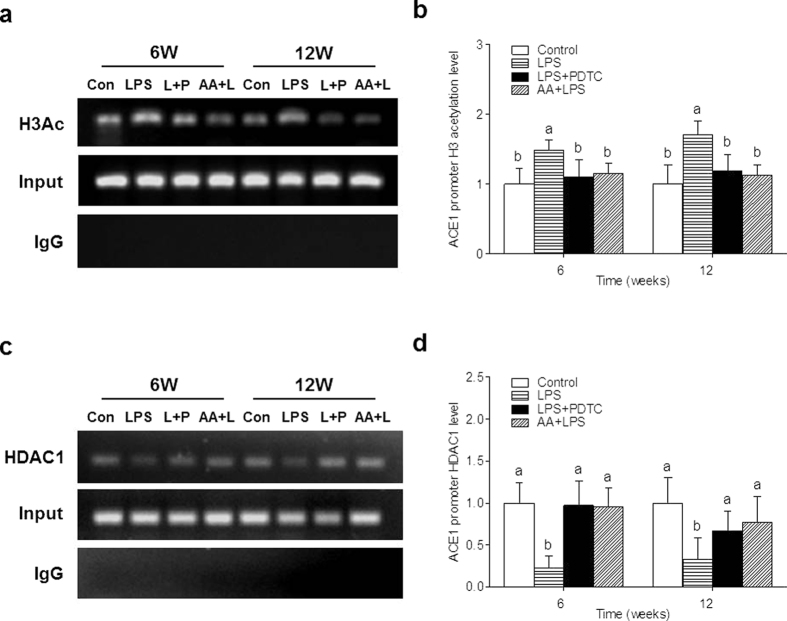
Effects of a prenatal LPS exposure and the PDTC or the AA treatment on renal cortex *ACE1* histone acetylation in offspring. (**a,b**) Renal cortex *ACE1* histone H3 acetylation was assessed by chromatin immunoprecipitation in 6 and 12 weeks old offspring. (**c,d**) Renal cortex *ACE1* HDAC1 was assessed by chromatin immunoprecipitation in 6 and 12 weeks old offspring. Data are expressed as mean ± SD. (n = 6 in each group). Different letters means significant differences (*P* < 0.05).

Therefore, we also focused on alteration of histone acetylation, which is another epigenetic modification in the *ACE1* promoter region. Compared with controls, the enrichment of *ACE1* promoter on histone 3 acetylation (H3Ac) in renal cortex showed a significant increase in the offspring of LPS group at 6 and 12 weeks. Furthermore, both PDTC and AA treatment observably decreased H3Ac enrichment on the *ACE1* promoter at 6 and 12 weeks (Fig. 5a,b). To understand the mechanism of the enrichment change of the *ACE1* promoter on H3Ac in renal cortex, we further detected the recruitments of histone deacetylase 1 (HDAC1) in the same region. Compared to controls, the enrichment of HDAC1 on the *ACE1* promoter had a significant decrease in the offspring of LPS group at 6 and 12 weeks. Additionally, both the PDTC and the AA treatment were found to elevate the enrichment of HDAC1 on the *ACE1* promoter at 6 and 12 weeks (Fig. 5c,d).

## Discussion

Hypertension is a major risk factor for cardiovascular and cerebrovascular disease. Environmental factors and genetic factors are two important factors for the development of hypertension, but it has also been assured that the later development of hypertension can be programmed by an adverse fetal environment^3^,^4^,^18^,^19^,^20^,^21^. Prenatal inflammation as a common event during pregnancy has been proven to be strongly associated with the hypertension in adult offspring^5^,^6^,^7^,^8^. Our group previously found that prenatal exposure to systemic inflammation induced by LPS stimulation led to hypertension, increased body weight, alteration of the intrarenal RAS, and renal damage in adult offspring rats^9^,^10^. These discoveries provide opportunities to develop new strategies and medical interventions for the prevention or the treatment of essential hypertension.

In this study, we successfully duplicated the model of prenatal exposure to LPS and found the increased body weight, SBP, and urinary protein level in the offspring of prenatal LPS exposure^9^,^10^,^22^. Based on our knowledge, our study is the first time to show that AA had the same protective effects on offspring’s elevated blood pressure and renal damages, like the PDTC treatment.

The renin-angiotensin system (RAS) is a hormonal cascade of biochemical reactions, which adjust the normal physiological function of the cardiovascular system^23^. Experimental evidence suggests that RAS plays an important role in fetal programming hypertension^24^. Local tissue *ACE1* plays a more critical role to regulate blood pressure than circulating *ACE1*^25^. Our current study found that the prenatal AA treatment reversed the elevated expression of *ACE1* at both the mRNA and protein levels, which also showed the effect similar to the prenatal PDTC treatment. Wang *et al*.^26^ showed intrarenal oxidative stress increased in offspring of prenatal LPS exposure. Surprisingly, prenatal AA also protects against intrarenal oxidative stress in offspring of prenatal LPS stimulation, such as the reduced MDA level and increased SOD and GSH activity as compared to that in offspring of prenatal LPS stimulation alone. All these finding suggested that prenatal AA may be a powerful weapon against the prenatal programmed hypertension.

Epigenetics refers to all meiotically and mitotically heritable changes in gene expression that are not coded in the DNA sequence itself^27^. Epigenetics plays an important role in gene expression regulation. Epigenetic mechanisms can convert environmental influences into changes in the expression of genes, and some studies show it has a remarkable role in brain development and the pathophysiology of neurodevelopmental disorders^28^,^29^,^30^. Furthermore, an increasing amount of evidence from animal studies supports the role of environmental epigenetics in fetal programming^18^,^31^,^32^,^33^,^34^. Epigenetic mechanisms mainly include DNA methylation, histone modification, as well as noncoding RNAs^35^. Among those, DNA methylation and histone modification constitute the major ways of epigenetic regulations^36^. DNA methylation usually involves in gene silencing, imprinting, and the suppression of retrotransposons^37^. Histone modification plays an important role in recruiting proteins that regulate overall chromatin structure^38^. Histone acetylation can change the structure of nucleosomes, make chromatin conformation in an open state that promote transcription factors binding to the chromosome, and then activate the transcription and express genes^39^. In this study, we found no significant changes in the DNA methylation of renal cortex *ACE1* promoter in offspring with prenatal LPS stimulation. In an interesting observation, Lee *et al*.^25^ demonstrated that *ACE1* is upregulated in local tissues of spontaneously hypertensive rats via histone code modifications. We thus focused on another epigenetic mechanism: histone modification. The H3Ac was significantly recruited in the *ACE1* promoter of offspring of LPS group at 6 and 12 weeks. Both PDTC and AA treatment observably decreased H3Ac enrichment on the *ACE1* promoter, which was consistent with its mRNA and protein expression. These evidences suggest that the increased *ACE1* expression is regulated at least in part by the augmentation of H3Ac on its promoter but not DNA methylation in the offspring rats of prenatal exposure to LPS. Further mechanistic studies revealed that the LPS treatment reduced HDAC1 enrichment, which is a component of the histone deacetylase complex, on the *ACE1* promoter. Additionally, the PDTC and the AA treatment also elevated HDAC1 enrichment in the same region. Therefore, it has been proposed that LPS may promote H3Ac recruit via repressing HDAC1 enrichment on the *ACE1* promoter, thereby increasing *ACE1* mRNA expression. The current study also found that prenatal AA and PDTC reversed the histone modification on the *ACE1* promoter. This is of great significance because several studies reported that fetal-programed diseases could pass to the next generation mainly through epigenetic modification^33^,^40^,^41^. Thus, we believe that AA as a routine supplement taken by pregnant women in the case of infection maybe prevent the prevalence of hypertension and related cardiovascular diseases.

In conclusion, we demonstrate that histone modification may be an important epigenetic factor that upregulates *ACE1* expression in renal cortex tissues of rat offspring with a prenatal LPS exposure. Our research also suggest that AA may have the protective effects on hypertension in prenatal inflammation-induced offspring. It decreases oxidative stress and normalizes *ACE1* expression through histone deacetylation of *ACE1* at the promoter region, and thereby reduces the blood pressure and renal damages in offspring. Taking ascorbic acid during pregnancy should be a new approach towards the prevention of fetal programming hypertension, and this is likely to have a large impact on reducing hypertension incidence and the cost of health care.

## Methods

### Animals

Nulliparous pregnant Sprague-Dawley rats were purchased from the Animal Centre of the Third Military Medical University (Chongqing, China). All animals were allowed free access to standard laboratory rat chow and tap water. Until parturition, pregnant rats were caged individually in a room at constant temperature (24 °C) and under a 12 h light/dark cycle. Pups were raised with a lactating mother until 4 weeks of age, and then they were removed to cages with four pups per cage.

The timed pregnant rats were randomly divided into four groups (n = 8 in each): a control group (Con), a LPS group (LPS), a LPS + PDTC group (L + P), and an AA + LPS group (AA + L). The rats in these groups were intraperitoneally administered 0.5 ml normal saline, 0.79 mg/kg LPS (Sigma, St Louis, MO, USA), 0.79 mg/kg LPS plus 100 mg/kg PDTC (Sigma), or 350 mg/kg AA plus 0.79 mg/kg LPS, respectively. LPS was given on gestational day 8, 10 and 12, whereas PDTC and AA was given daily from the day 8 to 14. The rats in the LPS group were given a normal saline injection on day 9, 11, 13 and 14, while rats in the control group were given normal saline daily during day 8 to 14.

Offspring were studied at the age of 4 to 12 weeks old. This study was conducted in accordance with the principles outlined in the National Institutes of Health Guide for the Care and Use of Laboratory Animals (http://grants1.nih.gov/grants/olaw/) and was approved by the local animal ethics committee at the Third Military Medical University.

### Body weight measurement

The body weights of offspring rats were regularly monitored once a week from the age of 4 to 12 weeks.

### Blood pressure measurement

Systolic blood pressure (SBP) was measured in eight conscious offspring rats in each group at 6, 8, 10 and 12 weeks of age using the standard tail-cuff method (ML125, Powerlab, AD Instruments, Castle Hill, Australia). Before measurement of SBP, rats were placed inside a warming chamber (about 34 °C) for 15 min and then placed in plastic restrainers. A cuff with a pneumatic pulse sensor was attached to the tail, and SBP was determined as described previously^10^. In each rat, mean SBP was calculated from three consecutive SBP recordings.

### Collection of urine, blood and kidney tissue

Offspring rats at 6 and 12 weeks of age were kept in metabolic cages for 24 h for urine collection when the rats were fed with standard rat chow. After anesthetized with urethane (20%), blood was collected by heart puncture. After decapitation, the kidney tissue was abscised and stored at −80 °C.

### Urinary protein measurement

Urinary protein level was determined using a spectrophotometric sequential injection method^42^.

### Real-time RT-PCR

The mRNA expression of angiotensin-converting enzyme 1 (*ACE1*), angiotensin II type-1a receptor (*AT1a*), angiotensin II type-1b receptor (*AT1b*), and angiotensin II type-2 receptor (*AT2*) in renal cortex tissue were assessed by real-time RT-PCR when the offspring were at 6 or 12 weeks old, according to a method previously described^9^. Total RNA was extracted from renal cortex using a RNAsimple Total RNA Kit (TIANGEN Biotech, Beijing, China). Total RNA (1 μg) was then reverse-transcribed into cDNA using a PrimeScript™ RT reagent kit with gDNA Eraser (TaKaRa Biotechnology, Dalian, China). *GAPDH* was used as an internal control. The PCR primers used were designed by Premier 5.0 (PREMIER Biosoft international, Palo Alto, CA, USA) based on the published nucleotide sequences and listed in Table 1. Each real-time PCR reaction was carried out in a total volume of 25 μl with SYBR^®^ Premix Ex Taq™ II (Tli RNaseH Plus) (TaKaRa Biotechnology) according to the following conditions: 30 s at 95 °C, 40 cycles at 95 °C for 15 s, 60 °C for 15 s, 72 °C for 20 s, using the Eppendorf Mastercycler ep realplex system (Eppendorf, Hamburg, Germany). The cycle threshold (Ct) values were normalized to the expression levels of *GAPDH*. The relative mRNA expression level of each gene was calculated using the equation 2^−ΔΔCt^.

### Western blot

Western blotting analyses were performed as previously described^9^. Total protein in renal cortex of rat offspring at 6 and 12 weeks old were extracted, and protein concentrations were measured by a Bicinchoninic acid kit (Beyotime Biotechnology, Shanghai, China). After denaturation, equal amounts of proteins (50 μg) were resolved through 10% SDS-PAGE and transferred onto nitrocellulose membranes. The membranes were blocked with 5% nonfat milk in TBST for 1 h at room temperature. After incubation with anti-ACE1 (1:1000, ab11734, Abcam, Cambridge, MA, USA), anti-AT1-R (1:2000, ab9391, Abcam), or anti-GAPDH (1:5000, 2118S, Cell Signaling Technology, Beverly, MA, USA) antibodies overnight at 4 °C, the membranes were incubated with a peroxidase-conjugated secondary antibody in TBST for 1 h at room temperature. The specific bands were detected by enhanced chemiluminescence and recorded on X-ray film. The Quantity One software (Bio-Rad, Hercules, CA, USA) was used to quantify the band intensities, and data were normalized to GAPDH levels.

### Evaluation the level of oxidative stress

To assess oxidative stress, serum and renal malondialdehyde (MDA) levels, a marker for lipid peroxidation, were determined using a MDA assay kit (TBA method) (Nanjing Jianchen Bioengineering Institute, Nanjing, China). To assess antioxidants, serum and renal samples from offspring rats were used to measure superoxide dismutase (SOD) activity using a total SOD assay kit (Hydroxylamine method) (Nanjing Jianchen Bioengineering Institute), and glutathione (GSH) levels, using a Reduced GSH assay kit (Nanjing Jianchen Bioengineering Institute) following the manufacturer’s instructions.

### Detection the CpG island DNA Methylation of *ACE1*

Genomic DNA was extracted from renal cortex tissue by using the High Pure PCR Template Preparation Kit (Roche Diagnostics, Mannheim, Germany). The EZ DNA Methylation Kit (Zymo Research, Orange, CA, USA) was used for bisulfite conversion of the genomic DNA. We designed primer for *ACE1* gene to cover the regions that were with the higher CpG islands by using EpiDesigner (Sequenom, San Diego, CA, USA). The selected amplicon was started from the promoter and ended in the first exon of the *ACE1* gene (−338 to + 108 bp, Fig. 4a). In PCR amplification, a T7-promoter tag was added to the reverse primer, and a 10-mer tag sequence was added to the forward primer to balance the PCR primer length. The primer sequences are described in Table 2. DNA methylation levels were determined using a MassARRAY Compact MALDI-TOF (Sequenom), and methylation data for individual units (1–3 CpG sites per unit) were analyzed using the EpiTYPER software v4.0 (Sequenom).

### Chromatin immunoprecipitation (ChIP) assay

ChIP analysis was carried out according to the manufacturer’s instructions with minor changes using EZ-Magna ChIP^TM^ A/G (Millipore, Billerica, MA, USA). Briefly, tissues were fixed with 1% formaldehyde and washed with ice cold 1 × PBS. After homogenization, tissues were incubated with cell lysis buffer for 30 min on ice and then nuclei lysis buffer for 20 min on ice. The lysate were sonicated with 15 cycles at 90% output control for 15 s, followed by cooling on ice for 1 min. After centrifugation, 50 μl of supernatant containing solubilized chromatin were diluted 10-fold with dilution buffer. For immunoprecipitation, the diluted chromatin solutions were incubated with anti-acetyl-histone H3 (5 μg, 06–599, Millipore) or anti-HDAC1 (2 μg, 17–10199, Millipore) antibodies overnight at 4 °C. The input DNAs were taken from diluted solutions before immunoprecipitation. The immunocomplex were harvested by protein A/G magnetic beads. Beads were collected by magnetic separator and washed serially with a low salt wash buffer, high salt wash buffer, LiCl wash buffer, and TE buffer. The immune complexes were eluted from the beads with ChIP elution buffer containing proteinase K. To reverse the formaldehyde cross-links and eliminate proteins, elutes were incubated at 62 °C for 4 h with shaking and then at 95 °C for 10 min. The DNAs were purified with spin columns. The promoter DNA fragments were amplified by real-time PCR, and the cycling conditions were as follows: 30 s at 95 °C, 40 cycles at 95 °C for 15 s, 60 °C for 15 s, 72 °C for 20 s, using the Eppendorf Mastercycler ep realplex system (Eppendorf, Hamburg, Germany). The cycle threshold (Ct) values were normalized by input. The primers used in ChIP assays are shown in Table 2 25. The region on the *ACE1* promoter detected by ChIP assays is depicted in Fig. 4a. The results of ChIP assays were detected by PCR and representative results were presented as gel bands, which were separated on 1.5% agarose gels with electrophoresis and were captured by the gel document system (Bio-Rad).

### Statistical analysis

Results were expressed as means ± standard deviation (SD). One-way ANOVA model followed by the Tukey’s post hoc test was used for multiple comparisons. P < 0.05 was considered statistically significant. All analyses were carried out with SPSS 18.0 (SPSS Inc., Chicago, IL, USA).

## Additional Information

**How to cite this article**: Wang, J. *et al*. Ascorbic Acid Protects against Hypertension through Downregulation of ACE1 Gene Expression Mediated by Histone Deacetylation in Prenatal Inflammation-Induced Offspring. *Sci. Rep.* **6**, 39469; doi: 10.1038/srep39469 (2016).

**Publisher's note:** Springer Nature remains neutral with regard to jurisdictional claims in published maps and institutional affiliations.

## Figures and Tables

**Table 1:** Primers used in Real-time PCR.

Gene	Forward primer (5′-3′)	Reverse primer (5′-3′)	Amplicon size (bp)
*ACE1*	CCACCGTTACCAGACAACTATCC	GCGTATTCGTTCCACAACACCT	116
*AT1a*	ATGAGCACGCTTTCTTACCG	GCTGCCCTGGCTTCTGTC	132
*AT1b*	CAGCCGTCATCTACCGAAAC	AAGGAAAGGGAACACGAAGC	143
*AT2*	TGGCTGTGGCTGACTTACTCC	TGCACATCACAGGTCCAAAGAG	96
*GAPDH*	GATTTGGCCGTATCGGAC	GAAGACGCCAGTAGACTC	278

**Table 2:** Primers used in methylation analysis and ChIP assay.

Gene	Primer	Sequence (5′-3′)	Amplicon size (bp)
*ACE1*	tag-Forward	aggaagagagGAGGGTTATGTTAAATTTTTTGGGT	446
	T7-Reverse	cagtaatacgactcactatagggagaaggctACAACAACAACAACAACAACAAC	
*ACE1*	chip-Forward	AAAGGCTTGGAGGGTTATGC	160
	chip-Reverse	TGGGTGGAAGGATGAGGTTA	

## References

[b1] Wang R. . Impact of hypertension on health-related quality of life in a population-based study in Shanghai, China. Public Health 123, 534–539, doi: 10.1016/j.puhe.2009.06.009 (2009). 19665154

[b2] Staessen J. A., Wang J., Bianchi G. & Birkenhager W. H. Essential hypertension. Lancet 361, 1629–1641, doi: 10.1016/S0140-6736(03)13302-8 (2003). 12747893

[b3] Barker D. J., Bull A. R., Osmond C. & Simmonds S. J. Fetal and placental size and risk of hypertension in adult life. BMJ 301, 259–262 (1990). 2390618 10.1136/bmj.301.6746.259 PMC1663477

[b4] Barker D. J. The fetal origins of adult hypertension. J Hypertens Suppl 10, S39–44 (1992). 1291655

[b5] Alexander B. T. & Ojeda N. B. Prenatal inflammation and the early origins of hypertension. Clin Exp Pharmacol Physiol 35, 1403–1404, doi: 10.1111/j.1440-1681.2008.05074.x (2008). 18983576

[b6] Samuelsson A. M. . Prenatal exposure to interleukin-6 results in hypertension and alterations in the renin-angiotensin system of the rat. J Physiol 575, 855–867, doi: 10.1113/jphysiol.2006.111260 (2006). 16825309 PMC1995698

[b7] Hao X. Q. . Prenatal exposure to inflammation induced by zymosan results in activation of intrarenal renin-angiotensin system in adult offspring rats. Inflammation 33, 408–414, doi: 10.1007/s10753-010-9199-y (2010). 20229032

[b8] Liao W. . Prenatal exposure to zymosan results in hypertension in adult offspring rats. Clin Exp Pharmacol Physiol 35, 1413–1418, doi: 10.1111/j.1440-1681.2008.05062.x (2008). 18983578

[b9] Hao X. Q. . Prenatal exposure to lipopolysaccharide alters the intrarenal renin-angiotensin system and renal damage in offspring rats. Hypertens Res 33, 76–82, doi: 10.1038/hr.2009.185 (2010). 19911002

[b10] Wei Y. L., Li X. H. & Zhou J. Z. Prenatal exposure to lipopolysaccharide results in increases in blood pressure and body weight in rats. Acta Pharmacol Sin 28, 651–656, doi: 10.1111/j.1745-7254.2007.00593.x (2007). 17439721

[b11] Messina S. . Nuclear factor kappa-B blockade reduces skeletal muscle degeneration and enhances muscle function in Mdx mice. Exp Neurol 198, 234–241, doi: 10.1016/j.expneurol.2005.11.021 (2006). 16410003

[b12] Muller D. N. . NF-κB Inhibition Ameliorates Angiotensin II-Induced Inflammatory Damage in Rats. Hypertension 35, 193–201, doi: 10.1161/01.hyp.35.1.193 (2000). 10642297

[b13] Sawada H. . A nuclear factor-kappaB inhibitor pyrrolidine dithiocarbamate ameliorates pulmonary hypertension in rats. Chest 132, 1265–1274, doi: 10.1378/chest.06-2243 (2007). 17934115

[b14] Beswick R. A. . Long-Term Antioxidant Administration Attenuates Mineralocorticoid Hypertension and Renal Inflammatory Response. Hypertension 37, 781–786, doi: 10.1161/01.hyp.37.2.781 (2001). 11230373

[b15] Rodriguez-Iturbe B. . Early and sustained inhibition of nuclear factor-kappaB prevents hypertension in spontaneously hypertensive rats. J Pharmacol Exp Ther 315, 51–57, doi: 10.1124/jpet.105.088062 (2005). 15951402

[b16] Cederberg J., Siman C. M. & Eriksson U. J. Combined treatment with vitamin E and vitamin C decreases oxidative stress and improves fetal outcome in experimental diabetic pregnancy. Pediatr Res 49, 755–762, doi: 10.1203/00006450-200106000-00007 (2001). 11385134

[b17] Chen Y. H. . Ascorbic acid protects against lipopolysaccharide-induced intra-uterine fetal death and intra-uterine growth retardation in mice. Toxicology 217, 39–45, doi: 10.1016/j.tox.2005.08.010 (2006). 16171921

[b18] Bogdarina I., Welham S., King P. J., Burns S. P. & Clark A. J. Epigenetic modification of the renin-angiotensin system in the fetal programming of hypertension. Circ Res 100, 520–526, doi: 10.1161/01.RES.0000258855.60637.58 (2007). 17255528 PMC1976252

[b19] Zandi-Nejad K., Luyckx V. A. & Brenner B. M. Adult hypertension and kidney disease: the role of fetal programming. Hypertension 47, 502–508, doi: 10.1161/01.HYP.0000198544.09909.1a (2006). 16415374

[b20] Vehaskari V. M., Aviles D. H. & Manning J. Prenatal programming of adult hypertension in the rat. Kidney Int 59, 238–245, doi: 10.1046/j.1523-1755.2001.00484.x (2001). 11135076

[b21] Vehaskari V. M. & Woods L. L. Prenatal programming of hypertension: lessons from experimental models. J Am Soc Nephrol 16, 2545–2556, doi: 10.1681/ASN.2005030300 (2005). 16049066

[b22] Gao M. . Prenatal exposure to lipopolysaccharide results in local RAS activation in the adipose tissue of rat offspring. PLoS One 9, e111376, doi: 10.1371/journal.pone.0111376 (2014). 25360670 PMC4216013

[b23] Rosenbaugh E. G., Savalia K. K., Manickam D. S. & Zimmerman M. C. Antioxidant-based therapies for angiotensin II-associated cardiovascular diseases. Am J Physiol Regul Integr Comp Physiol 304, R917–928, doi: 10.1152/ajpregu.00395.2012 (2013). 23552499 PMC3680755

[b24] Mizuno M., Lozano G., Siddique K., Baum M. & Smith S. A. Enalapril attenuates the exaggerated sympathetic response to physical stress in prenatally programmed hypertensive rats. Hypertension 63, 324–329, doi: 10.1161/HYPERTENSIONAHA.113.02330 (2014). 24191284 PMC3891399

[b25] Lee H. A. . Tissue-specific upregulation of angiotensin-converting enzyme 1 in spontaneously hypertensive rats through histone code modifications. Hypertension 59, 621–626, doi: 10.1161/HYPERTENSIONAHA.111.182428 (2012). 22311897

[b26] Wang X. . Prenatal lipopolysaccharide exposure results in dysfunction of the renal dopamine D1 receptor in offspring. Free Radic Biol Med 76, 242–250, doi: 10.1016/j.freeradbiomed.2014.08.010 (2014). 25236748 PMC6873924

[b27] Wang X. . A genome-wide methylation study on essential hypertension in young African American males. PLoS One 8, e53938, doi: 10.1371/journal.pone.0053938 (2013). 23325143 PMC3542324

[b28] MacDonald J. L. & Roskams A. J. Epigenetic regulation of nervous system development by DNA methylation and histone deacetylation. Prog Neurobiol 88, 170–183 (2009). 19554713 10.1016/j.pneurobio.2009.04.002

[b29] Urdinguio R. G., Sanchez-Mut J. V. & Esteller M. Epigenetic mechanisms in neurological diseases: genes, syndromes, and therapies. Lancet Neurol 8, 1056–1072, doi: 10.1016/S1474-4422(09)70262-5 (2009). 19833297

[b30] Zhao X., Pak C., Smrt R. D. & Jin P. Epigenetics and Neural developmental disorders: Washington DC, September 18 and 19, 2006. Epigenetics 2, 126–134 (2007). 17965627 10.4161/epi.2.2.4236 PMC2700626

[b31] Maccani M. A. & Marsit C. J. Epigenetics in the placenta. Am J Reprod Immunol 62, 78–89, doi: 10.1111/j.1600-0897.2009.00716.x (2009). 19614624 PMC2813777

[b32] Fowden A. L. & Forhead A. J. Hormones as epigenetic signals in developmental programming. Exp Physiol 94, 607–625, doi: 10.1113/expphysiol.2008.046359 (2009). 19251980

[b33] Jirtle R. L. & Skinner M. K. Environmental epigenomics and disease susceptibility. Nat Rev Genet 8, 253–262, doi: 10.1038/nrg2045 (2007). 17363974 PMC5940010

[b34] Xita N. & Tsatsoulis A. Fetal origins of the metabolic syndrome. Ann N Y Acad Sci 1205, 148–155, doi: 10.1111/j.1749-6632.2010.05658.x (2010). 20840267

[b35] Bernstein B. E., Meissner A. & Lander E. S. The mammalian epigenome. Cell 128, 669–681, doi: 10.1016/j.cell.2007.01.033 (2007). 17320505

[b36] Yoon Y. S., Choo J. H., Yoo T., Kang K. & Chung J. H. RhoB is epigenetically regulated in an age- and tissue-specific manner. Biochem Biophys Res Commun 362, 164–169, doi: 10.1016/j.bbrc.2007.08.002 (2007). 17706596

[b37] Bird A. DNA methylation patterns and epigenetic memory. Genes Dev 16, 6–21, doi: 10.1101/gad.947102 (2002). 11782440

[b38] Strahl B. D. & Allis C. D. The language of covalent histone modifications. Nature 403, 41–45, doi: 10.1038/47412 (2000). 10638745

[b39] An W. Histone acetylation and methylation: combinatorial players for transcriptional regulation. Subcell Biochem 41, 351–369 (2007). 17484136

[b40] Barouki R., Gluckman P. D., Grandjean P., Hanson M. & Heindel J. J. Developmental origins of non-communicable disease: implications for research and public health. Environ Health 11, 42, doi: 10.1186/1476-069X-11-42 (2012). 22715989 PMC3384466

[b41] Walker C. L. & Ho S. M. Developmental reprogramming of cancer susceptibility. Nat Rev Cancer 12, 479–486, doi: 10.1038/nrc3220 (2012). 22695395 PMC3820510

[b42] Kanchana W. I., Sakai T., Teshima N., Katoh S. & Grudpan K. Successive determination of urinary protein and glucose using spectrophotometric sequential injection method. Anal Chim Acta 604, 139–146, doi: 10.1016/j.aca.2007.10.010 (2007). 17996535

